# AI-driven dynamic psychological measurement: correcting university student mental health scales using daily behavioral and cognitive data

**DOI:** 10.3389/fdgth.2025.1615250

**Published:** 2025-11-28

**Authors:** B. G. Tong, Zihong Liang, Xuemei He, Fan Yang, Li Yang, Lijia Gao

**Affiliations:** 1 Department of Psychiatry, Inner Mongolia People’s Hospital, Hohhot, China; 2 Student Management Department, Inner Mongolia People’s Hospital, Hohhot, China; 3 Inner Mongolia Clinical College, Inner Mongolia Medical University, Hohhot, China

**Keywords:** dynamic psychological measurement, artificial intelligence, scale correction, university student mental health, behavioral and cognitive data

## Abstract

**Objective:**

This study aimed to evaluate an Artificial Intelligence (AI)-driven dynamic psychological measurement method for correcting traditional mental health scales. We sought to validate its feasibility using daily behavioral and cognitive data from university students and assess its potential as an intervention tool.

**Methods:**

A total of 177 university students participated in a one-and-a-half-year study. Using a WeChat mini-program, we collected data from cognitive voting (87 instances), behavioral check-ins (66 instances), and standardized psychological scales (SAS, SDS, SCL-90). Scale scores were dynamically adjusted using Large Language Models (LLMs) and Retrieval-Augmented Generation (RAG) techniques. Paired-sample *t*-tests, MANOVA, and Cohen’s *d* were used to compare the performance of the dynamic model against traditional scales. Intervention effects were validated using the Hamilton Anxiety Rating Scale (HAM-A) and Hamilton Depression Rating Scale (HAM-D).

**Results:**

The dynamic assessment demonstrated superior performance in identifying both anxiety (SAS: dynamic model AUC = 0.95 vs. traditional AUC = 0.86) and depression (SDS: dynamic model AUC = 0.93 vs. traditional AUC = 0.82). Over three semesters, participating students showed significant decreases in clinically-rated anxiety scores on the HAM-A (15.2% reduction; 95% CI for mean difference [1.00, 5.25], *p* = 0.004) and depression scores on the HAM-D (40.0% reduction; 95% CI for mean difference [2.71, 7.71], p<0.001). High student engagement was observed (cognitive voting participation: 79%; behavioral check-ins: 42%). While the dynamic adjustment for the SCL-90 was initially effective ($R^{2}=0.34$), its specificity later decreased, potentially due to interference from life factors (dynamic model MSE = 102.74 vs. traditional MSE = 84.17).

**Discussion:**

AI-driven dynamic assessment provides superior accuracy for anxiety (SAS) and depression (SDS) scales over static methods by effectively capturing psychological fluctuations. The significant reductions in clinically-rated anxiety and depression suggest the system may function as an integrated assessment-intervention loop, fostering self-awareness through continuous feedback. High user engagement confirms the method’s feasibility. However, the model’s diminished specificity for the complex SCL-90 scale over time highlights challenges in handling intricate, long-term symptom patterns. This research supports a shift towards continuous “digital phenotyping” and underscores the need for rigorous validation, multimodal data integration, and robust ethical considerations.

## 1 Introduction

### 1.1 Background

The field of psychometrics faces a fundamental challenge rooted in the philosophical nature of mental states: the inherent tension between stable, long-term traits and fluctuating, moment-to-moment psychological states (1). Traditional psychometric assessments, while valuable, often provide a static “snapshot” of an individual’s mental health, which may not adequately capture the dynamic, real-time fluctuations of affective states such as anxiety and depression. This is particularly critical for university students, a demographic navigating a period of significant developmental and academic pressure. Recent large-scale epidemiological studies in China have highlighted the severity of this issue, with detection rates for depression and anxiety among university students reported at 9.8% and 15.5%, respectively (2). The mental health landscape for medical students can be even more demanding, characterized by a rigorous curriculum and high-stakes examinations, necessitating more sensitive and ecologically valid assessment methods (3). Consequently, accurately capturing the ebb and flow of **state anxiety** and **state depression** remains a critical research topic.

Within psychological research, measurement is broadly approached through observational, experimental, and psychometric testing methods (4). While observational and experimental methods offer high validity, their resource-intensive nature limits scalability. Psychometric testing, conversely, offers convenience and has been widely adopted. However, this convenience often comes at the cost of ecological validity, as a single questionnaire provides limited insight into a student’s psychological journey. This limitation reveals a significant gap: the need for a measurement paradigm that integrates the scalability of psychometric tests with the contextual richness of observational methods. To truly understand and potentially “correct” a student’s self-reported score, it is essential to look beyond the score itself and examine the underlying cognitive and behavioral patterns that drive these emotional states.

This perspective aligns with the principles of **Cognitive-Behavioral Theory (CBT)**, which posits that emotions are shaped by an interplay of thoughts and behaviors. The continuous advancement of Information and Communication Technology (ICT) and Artificial Intelligence (AI) now offers an unprecedented opportunity to operationalize this holistic view at scale. The field is already witnessing significant progress through innovations like Computerized Adaptive Testing (CAT) (5, 6) and Automatic Item Generation (AIG) (7). The ubiquity of smartphones has further enabled the collection of rich, longitudinal data via social networks, allowing for the creation of dynamic user profiles that integrate network theory with psychological process models (8).

More recently, the emergence of powerful AI, particularly large language models like ChatGPT, is poised to act as a “human proxy” in interpreting complex behavioral data, heralding a quiet revolution in psychometrics (9, 10). The trajectory of psychological measurement is inevitably shifting towards capturing multimodal data from daily life via wearable devices—including facial expressions, voice patterns, and even brain imaging scans—long before Brain-Computer Interfaces (BCIs) might achieve true “mind-reading” (11). Crucially, this technological evolution is blurring the boundary between mental health assessment and clinical intervention (12). Modern intelligent applications are evolving from passive monitors into “digital health promoters,” capable of providing data-driven, adaptive interventions based on real-time psychometric insights (13, 14). These systems create a bidirectional feedback loop, where assessment informs personalized intervention, and the resulting behavioral changes continuously update the assessment itself. Such detailed personal profiles not only guide psychological interventions (15) but also serve as core indicators for evaluating their effectiveness and tracking mental health status over time (16). This technologically-empowered, integrated paradigm forms the foundation for the novel assessment framework proposed in this study.

### 1.2 Research objectives

Against the backdrop of the inherent challenges in traditional psychometrics and the opportunities afforded by technological innovation, this research aims to pioneer and implement a novel pathway for university student mental health assessment. Utilizing a WeChat mini-program platform, we deeply integrate information technology with psychological theory. Drawing inspiration from the principles of Ecological Momentary Assessment (EMA), which emphasize capturing experiences in real-time, real-world contexts (17), we constructed a dynamic, interactive, and continuous mental health assessment ecosystem.

The core objectives of this study were threefold. First, we aimed to **evaluate the accuracy** of an AI-driven dynamic assessment model, testing whether integrating daily cognitive and behavioral data could correct and enhance the performance of traditional static mental health scales. Second, we sought to **explore the potential intervention effect** of the system, investigating whether continuous engagement and feedback could lead to measurable improvements in students’ clinically-rated mental health. Third, we intended to **assess the feasibility and user engagement** of this approach, examining its practicality and acceptance within a real-world university setting.

To achieve these objectives, our conceptual strategy was to create a multidimensional, multimodal mental health assessment framework. This framework creatively integrates three distinct data streams: (1) traditional, standardized psychometric scales for baseline assessment; (2) motivation-driven “cognitive voting” to capture students’ thought tendencies and emotional responses to daily events (18); and (3) systematic “behavioral check-ins” for the idiographic measurement of actual behavioral patterns (19). By continuously collecting and integrating these data points, our system aims to construct dynamic, real-time mental health profiles for each student.

Ultimately, the goal of this study extends beyond passive assessment. By providing immediate feedback and gamifying personality attributes, we aim to actively engage students in their own mental health management. This process is designed to foster self-awareness and encourage positive behavioral or cognitive adjustments, thereby creating an integrated assessment and intervention loop. In essence, this research endeavors to develop and validate an intelligent and personalized tool that is more attuned to the contemporary needs of university mental health services, achieving greater immediacy, comprehensiveness, and personalization (20).

## 2 Methods

### 2.1 Overview

This study employed a longitudinal design to dynamically assess the psychological states of 177 undergraduate clinical medicine students over one and a half years. The research was conducted via a custom-developed WeChat mini-program, which served as the primary platform for data collection and user interaction. Our methodological framework integrated three core data streams: (1) periodic, systematic self-report scale screenings conducted at the beginning of each semester; (2) high-frequency ecological cognitive voting, comprising 87 instances distributed throughout the study period; and (3) event-driven behavioral check-ins, with 66 distinct instances recorded over the same period.

The core self-report scales included the Self-Rating Anxiety Scale (SAS), the Self-Rating Depression Scale (SDS), the Symptom Checklist-90 (SCL-90), the Eysenck Personality Questionnaire (EPQ), the Sixteen Personality Factor Questionnaire (16PF), and the Myers-Briggs Type Indicator (MBTI). Cognitive voting was designed as a method for students to express opinions and emotional attitudes regarding their campus life and current events through multiple-choice questions. Behavioral check-ins involved the systematic recording of specific events each student encountered, such as academic achievements, social conflicts, or personal milestones. These events were defined and logged by four designated “life teachers” (counselors), providing a semi-objective measure of students’ real-world experiences.

### 2.2 Measures and instruments

A battery of standardized psychometric scales was administered to collect baseline and periodic data on students’ mental health status. All selected instruments have been validated for use within the Chinese cultural context. The internal consistency for these scales, as reported in validation studies with Chinese university students or related populations, is detailed below.

- **Self-rating anxiety scale (SAS).** The SAS is a 20-item self-report questionnaire used to measure the subjective severity of anxiety symptoms. The Chinese version of the SAS, introduced and validated by Wang (1984), has demonstrated good psychometric properties in Chinese populations (21). Subsequent validation studies among university students have reported high internal consistency, with Cronbach’s alpha coefficients typically around 0.88 (22).
- **Self-rating depression scale (SDS).** The SDS is a 20-item self-report instrument designed to assess the level of depressive symptomatology. The Chinese version has been widely used and validated (21). Studies have confirmed its reliability and validity among various Chinese populations, including university students, with reported Cronbach’s alpha values typically ranging from 0.84 to 0.89 (23, 24).
- **Symptom checklist-90 (SCL-90).** The SCL-90 is a comprehensive 90-item self-report inventory that evaluates a broad range of psychological problems and symptoms of psychopathology across nine primary symptom dimensions. The Chinese version was introduced by Wang (1984) and its utility in the Chinese context has been extensively confirmed, with excellent internal consistency (Cronbach’s alpha often > 0.90) reported in validation studies (21, 25).
- **Eysenck personality questionnaire (EPQ).** The EPQ is a self-report questionnaire designed to measure major personality dimensions: Psychoticism (P), Extraversion (E), and Neuroticism (N), along with a Lie (L) scale. We utilized the Chinese revised version developed by Gong (1986), which is well-validated for Chinese adults (26). Its subscales have shown acceptable to good reliability, with Cronbach’s alpha coefficients generally ranging from 0.70 to 0.85 (27).
- **Sixteen personality factor questionnaire (16PF).** The 16PF is a comprehensive personality assessment tool that measures 16 primary personality factors. The Chinese revised version has been validated and normed for the Chinese population, including university students (28, 29). The reliability for its various factors typically falls within an acceptable range for personality measures (Cronbach’s alpha: 0.60–0.80).
- **Myers-Briggs type indicator (MBTI).** The MBTI is a self-report questionnaire indicating different psychological preferences in how people perceive the world and make decisions. In this study, the MBTI was not used as a rigid diagnostic or predictive tool, but rather as an auxiliary instrument to facilitate student engagement and self-exploration. The personality type descriptions served as a dynamic “character profile” within the mini-program, providing a basis for the AI to generate personalized feedback and gamified interactions, thereby transforming static scores into a narrative, humanized experience. We referenced the Chinese version of the instrument, which has demonstrated adequate reliability (Cronbach’s alpha: 0.70–0.85) in validation studies (30).

### 2.3 Study design and procedure

This study employed a longitudinal design combining principles of psychometric testing, Ecological Momentary Assessment (EMA), and digital intervention. Over a one-and-a-half-year period, we conducted a dynamic assessment of psychological states involving 177 undergraduate clinical medicine students. The entire study was facilitated through a custom-developed WeChat mini-program, which served as the primary interface for data collection, feedback delivery, and student engagement. The research procedure involved an initial baseline assessment using standardized scales at the beginning of the first semester, followed by continuous data collection through weekly cognitive voting and event-contingent behavioral check-ins, alongside periodic reassessments with the full scale battery at the start of subsequent semesters.

### 2.4 The dynamic assessment framework

To derive both quantitative and qualitative insights from the rich, multimodal data, we developed a novel conceptual framework to structure our assessment. This framework was designed to bridge the gap between high-level psychometric scores and the granular, real-world data collected daily. Drawing inspiration from psychometric modeling approaches such as the bifactor model (31), testlet models (32), and second-order factor models (33), as well as the time-series nature of Vector Autoregression (VAR) models (34), we established an intermediate hierarchical structure. This collaborative effort, involving four experienced university life teachers and three clinical psychologists, resulted in a model that could systematically capture and interpret the psychological and behavioral patterns reflected in students’ daily lives.

The structure consists of six primary factors: Anxiety Cognition, Anxiety Behavior, Depression Cognition, Depression Behavior, Personality Cognition, and Personality Behavior. Each primary factor was further subdivided into four sub-dimensions (e.g., negative events, developmental concerns, life discipline), resulting in a total of 24 cognitive and behavioral dimensions. The complete framework is detailed in Table 1.

**Table 1:** Cognitive and behavioral dimensions and their relationship with psychometric scales.

Dimensions	Anxiety cognition	Anxiety behavior	Depression cognition	Depression behavior	Personality cognition	Personality behavior
Negative events	Pessimistic outlook	Excessive avoidance	Negative self-assessment	Somatization	Extreme notions	Defense mechanisms
Developmental concerns	Excessive worry	Neuroticism	Lack of emotional expression	Hedonistic withdrawal	Values	Paranoid behavior
Life discipline	Procrastination	Compulsion	Passive withdrawal	Absenteeism or tardiness	Philosophy of life	Individuality
Interpersonal interaction	Over-sensitivity	Stressful events	Social isolation	Negative events	Hostile margins	Interpersonal conflict events

**Figure 1:**
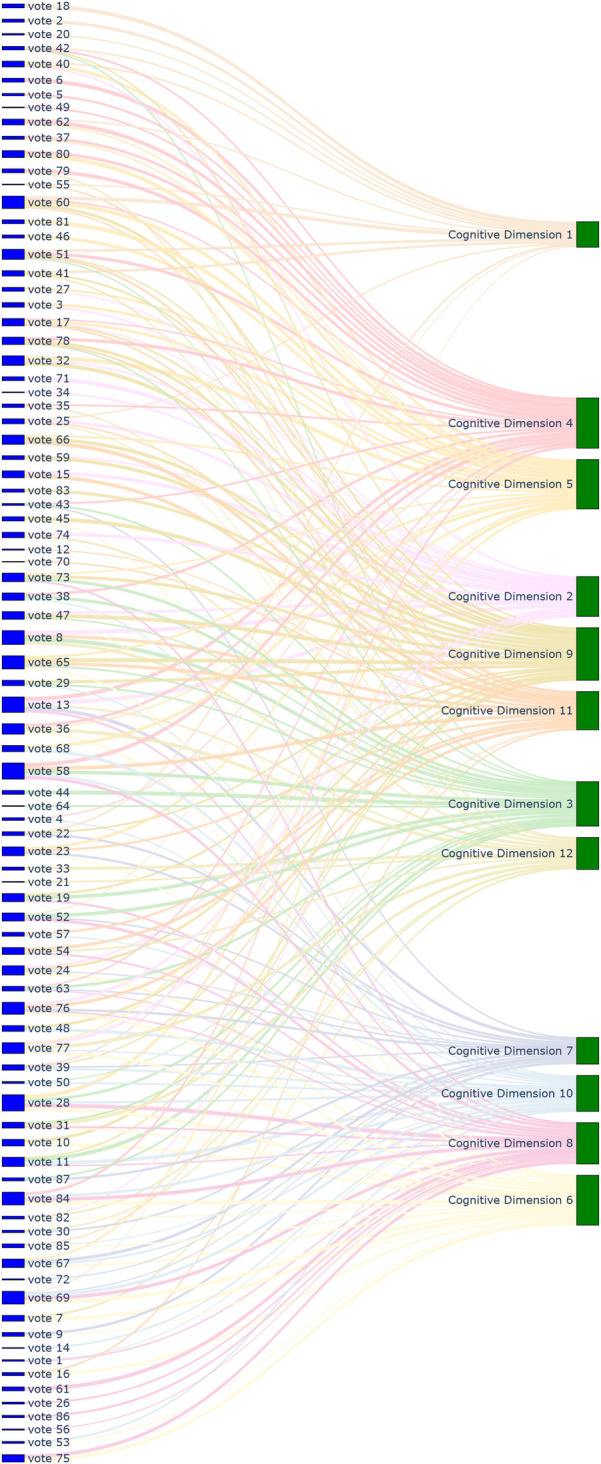
Sankey diagram mapping 87 cognitive voting results to 12 cognitive dimensions.

**Figure 2:**
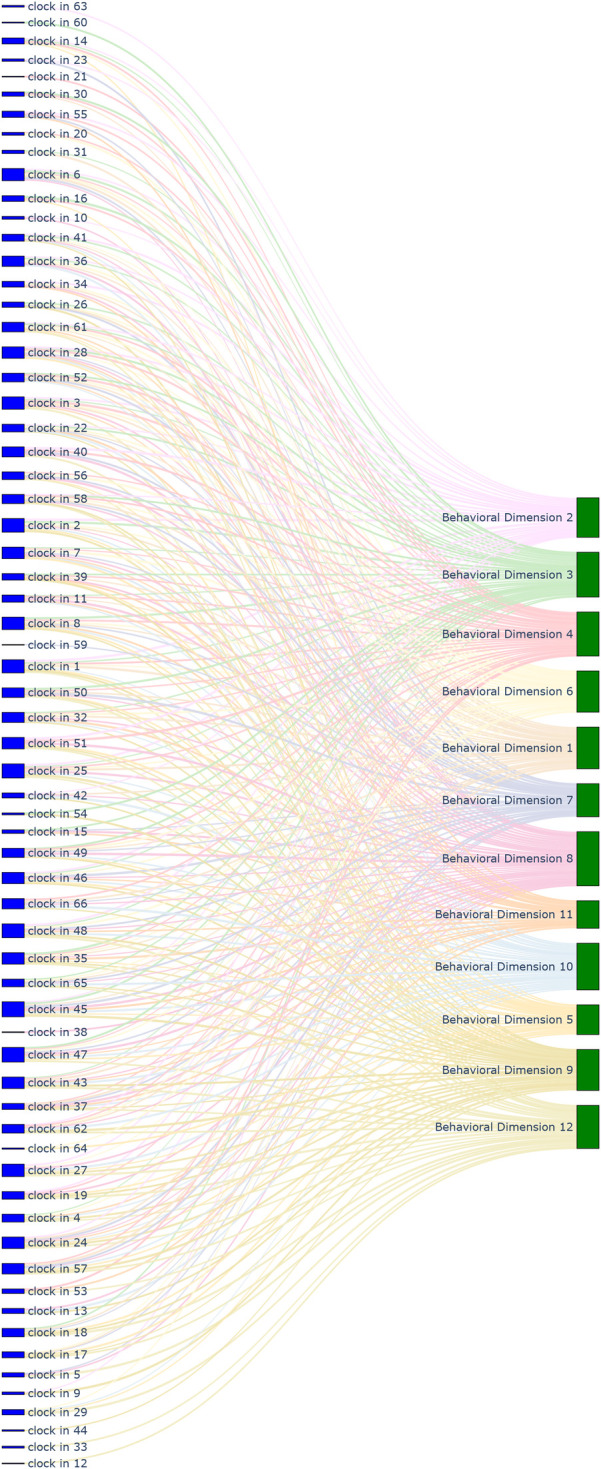
Sankey diagram mapping 66 behavioral check-in records to 12 behavioral dimensions.

**Figure 3:**
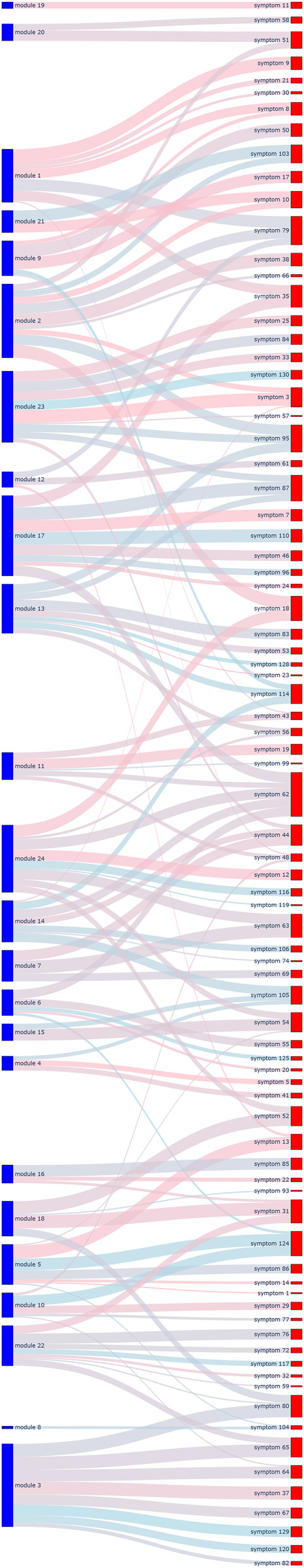
Sankey diagram mapping 24 cognitive and behavioral dimensions to 130 symptom scale items.

**Figure 4:**
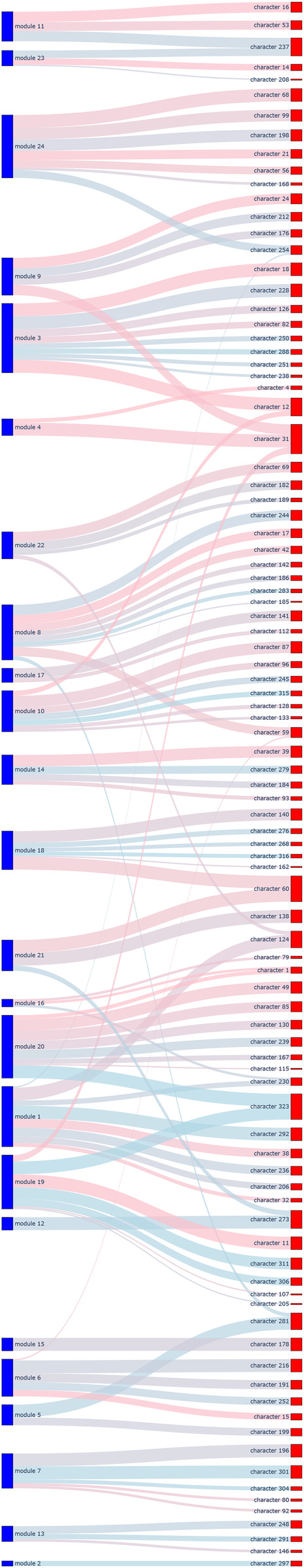
Sankey diagram mapping 24 cognitive and behavioral dimensions to 328 personality assessment items.

This framework served two key purposes. First, it provided a structured schema to ensure that every designed cognitive vote and behavioral check-in could be systematically mapped to one or more specific dimensions. At the outset of the study, we also mapped all items from the psychometric tests to these 24 dimensions. Specifically, the 130 items from SAS, SDS, and SCL-90 were linked to the framework, primarily for weighting their original scores during the assessment process. The 328 items from EPQ, 16PF, and MBTI were also linked, serving as a reference for personality assessment and allowing students to observe continuous changes in their profiles. This entire mapping process (visualized in Figures 1–4, which are detailed later) created a comprehensive, interconnected data ecosystem.

Second, the framework acted as a reference for the dynamic adjustment algorithm. For instance, if a student repeatedly selected pessimistic options in cognitive voting, their scores on the SDS and related SCL-90 dimensions would be algorithmically adjusted. Similarly, demonstrated self-discipline in behavioral check-ins would influence their personality trait profiles derived from the MBTI and 16PF, which are understood to be shaped by cognitive development and cultural adaptation processes during university years (35). These traits, in turn, are predictive of significant life outcomes (36). This continuous, iterative design and adjustment process aimed to produce a psychological status description that more closely approximates the reality captured by observational methods.

### 2.5 Application of AI models

To enhance assessment efficiency and accuracy, our framework leveraged a combination of Large Language Models (LLMs) and Retrieval-Augmented Generation (RAG) techniques. This AI-driven approach automated and refined the process of linking ecological data to psychometric constructs. The specific commercial LLM API utilized was Alibaba’s Qwen series (model: qwen-max).

**Figure 5:**
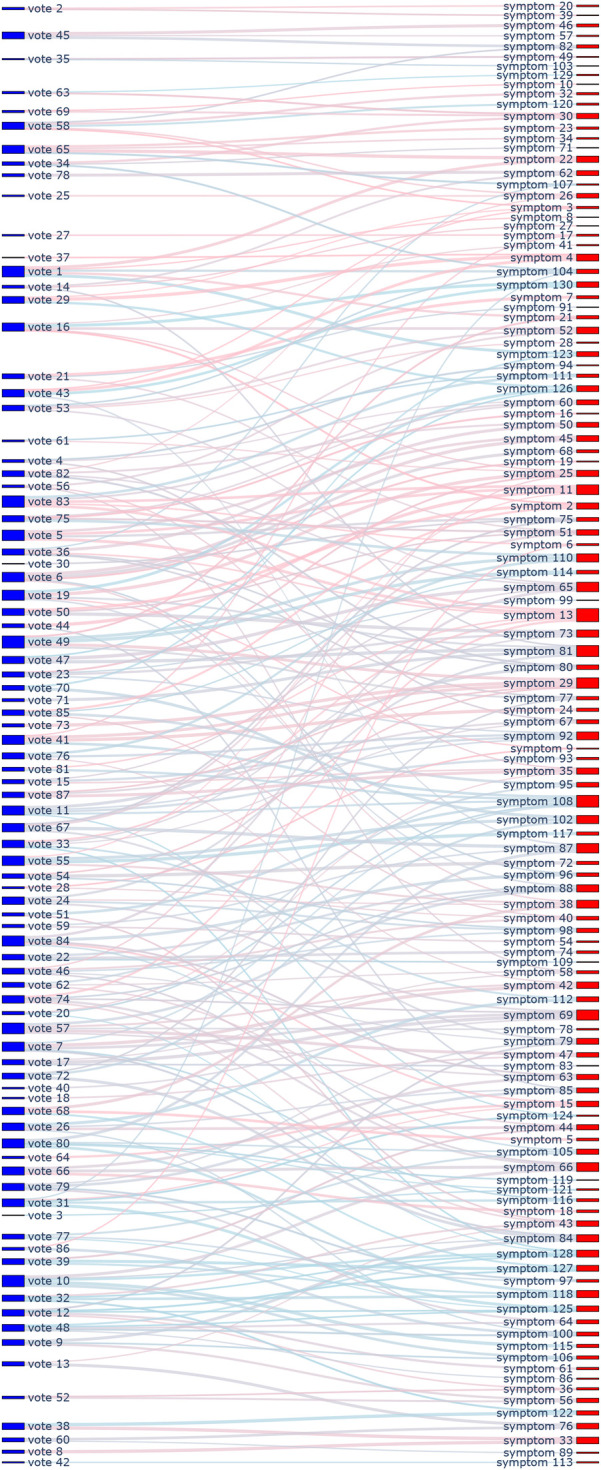
Sankey diagram mapping 87 cognitive voting results to 130 symptom scale items.

**Figure 6:**
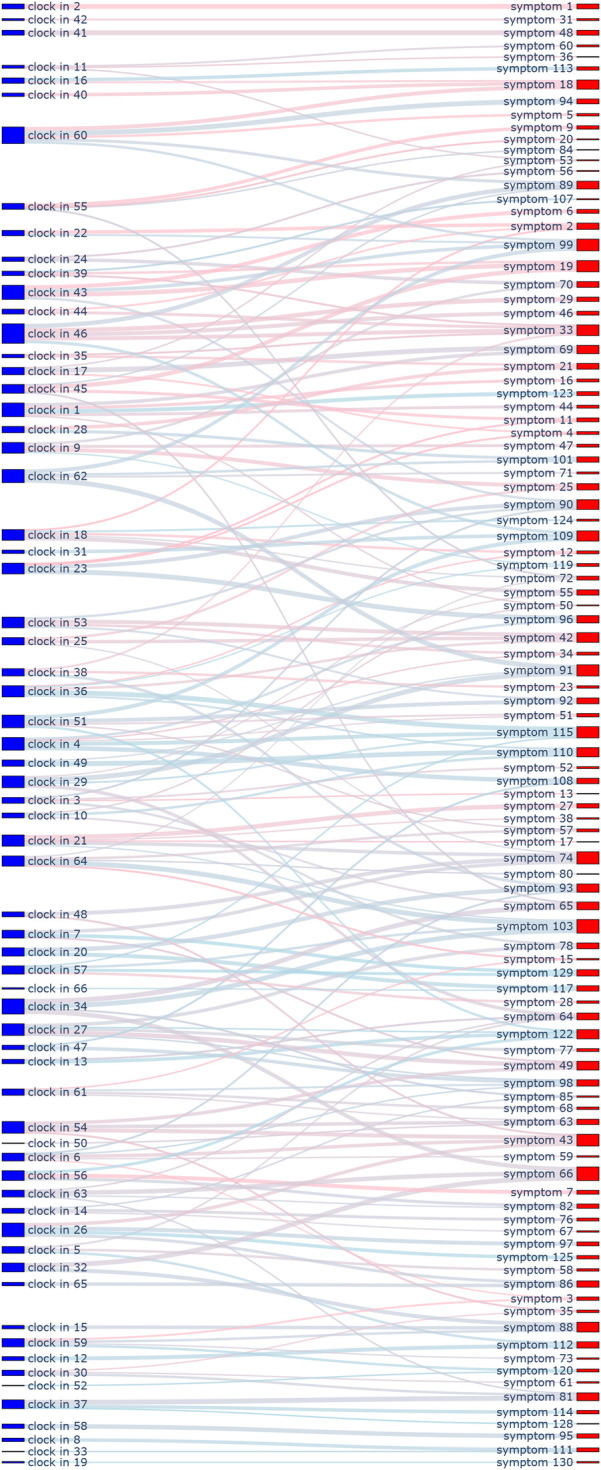
Sankey diagram mapping 66 behavioral check-in records to 130 symptom scale items.

**Figure 7:**
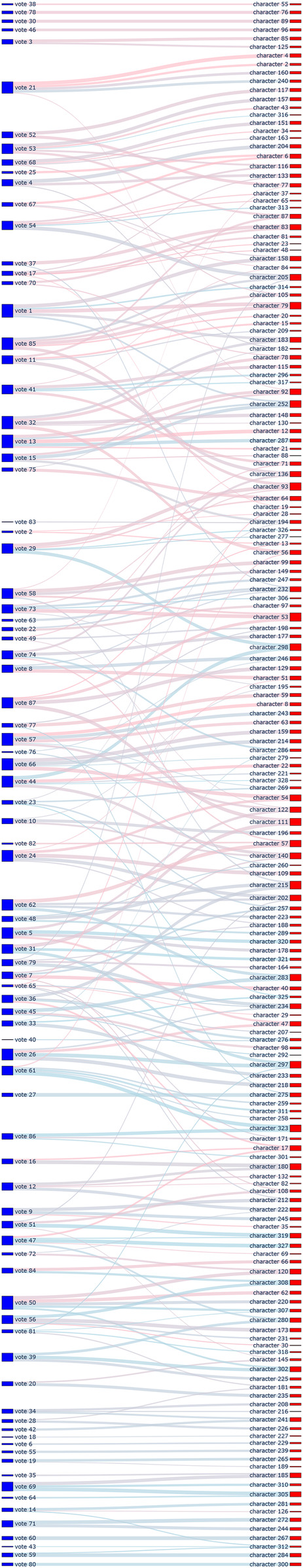
Sankey diagram mapping 87 cognitive voting results to 328 personality assessment items.

**Figure 8:**
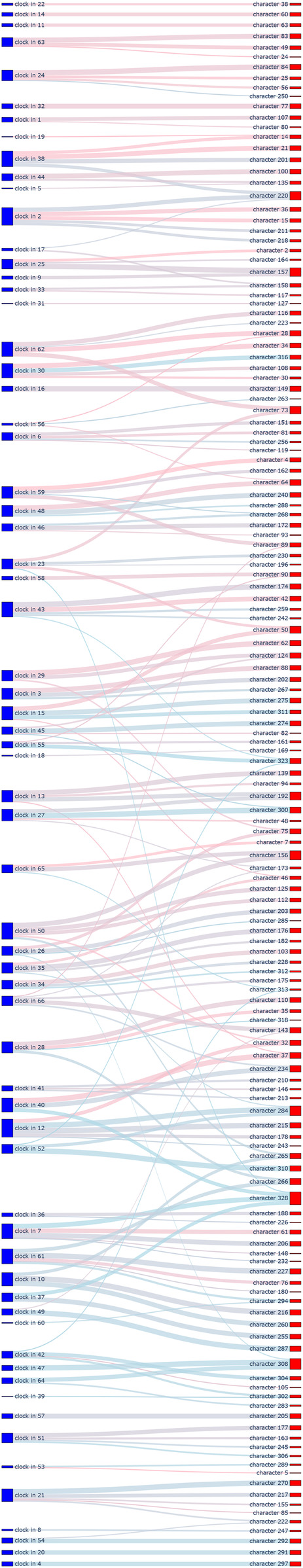
Sankey diagram mapping 66 behavioral check-in records to 328 personality assessment items.

The application of AI models involved a multi-stage process:

1. **Automated content mapping:** The LLM played a crucial role in assisting our research team (comprising life teachers and clinical psychologists) in the initial design and ongoing management of the ecological assessment content. Specifically, it was used to automate the mapping of each cognitive voting item and behavioral check-in definition to the 24 dimensions of our conceptual framework. This process significantly enhanced the standardization and efficiency of our content management. Through the use of the LLM, mapping tasks that would have required hours of manual expert deliberation were completed in minutes, greatly improving the real-time scalability of the assessment. The results of this comprehensive mapping are visualized in Figures 1–8.
2. **Retrieval-augmented generation (RAG) for dynamic adjustment:** To perform dynamic adjustments on student scores and profiles, we implemented a RAG model. The knowledge base for this model was constructed from three distinct sources: (1) **Personal Information,** including students’ historical behavioral data, academic records, and extracurricular activities, provided by counselors; (2) **Psychological Knowledge,** a text library containing principles of Cognitive-Behavioral Theory, personality trait descriptions, and excerpts from key psychometric textbooks; and (3) **Scale Scoring Protocols,** a structured database of all items, dimensions, and scoring rules for the psychometric scales used.
  When new data was received (e.g., a student’s response to a cognitive vote or a free-text comment), the RAG system would first retrieve the most relevant information from this comprehensive knowledge base. This retrieved context, which might include the student’s recent behaviors, relevant personality traits, and specific item-weighting rules, was then fed to the LLM.
3. **Personalized feedback generation:** Based on the context provided by the RAG process, the LLM then performed two key functions. First, it calculated the necessary adjustments to the student’s scores on relevant scales (e.g., SAS, SDS, SCL-90). Second, and perhaps more importantly, it generated personalized, narrative-based feedback. For example, a change in a student’s MBTI profile based on recent data would trigger the LLM to generate a descriptive text explaining this shift in a humanized, supportive tone, transforming a simple score change into a meaningful “character story” for the student.

This integrated AI approach not only streamlined the complex process of linking daily life data to psychometric scores but also enabled the delivery of dynamic, personalized, and narrative-rich feedback, which was central to the intervention aspect of our study design.

### 2.6 Gamified intervention and engagement

A core tenet of our study design was to move beyond passive measurement and actively promote students’ mental health through a multi-faceted, interactive environment. The mini-program was designed not only to evaluate psychological states but also to foster self-awareness and motivate positive change. This was achieved by integrating gamified feedback, community interaction, and a nuanced alert mechanism, all delivered within the students’ everyday life contexts (37).

#### 2.6.1 Gamified feedback and personality development

Instead of presenting students with raw anxiety or depression scores, we utilized trait descriptions from personality scales (e.g., EPQ, 16PF, MBTI) to create dynamic attributes, akin to those of a virtual character. When students participated in cognitive voting or completed behavioral check-ins, their actions had direct consequences on their “character profile,” earning them experience points and leading to attribute changes. For example, consistently choosing proactive behaviors could increase a “conscientiousness” attribute. These changes were instantly reflected in their personalized profile, providing immediate, narrative-based feedback. This gamified approach was intended to motivate students’ intrinsic drive to improve their own psychological state by adjusting their daily behaviors or cognitive patterns.

#### 2.6.2 Community module and personalized rankings

To further enhance engagement, we designed a community module that provided interactive, personalized rankings. These rankings did not display raw symptom scores. Instead, they reflected psychological fluctuations in relation to recent campus events (e.g., exams, social activities), using contextual descriptions (e.g., “recent obsessive thinking” or “hostile emotions”) rather than clinical labels. This allowed students to compare their psychological state with that of their peers in a de-stigmatized manner, motivating them to make positive adjustments when they noticed significant deviations.

#### 2.6.3 Alert mechanism and manual intervention

The system incorporated a multi-layered alert mechanism. For the administrative end (life teachers and counselors), the system provided direct notifications when a student’s dynamically adjusted symptom scores (from SAS, SDS, or specific SCL-90 dimensions) exceeded predefined clinical thresholds, prompting timely manual intervention.

For the student-facing end, the alert was more nuanced. When scores reached a warning level, the AI would generate descriptive feedback highlighted in red. This feedback would not simply state a high score, but would translate it into a context-aware, narrative warning. For instance, for a student with an INFP personality profile whose paranoia subscale score increased, the system might generate a message suggesting they may be feeling overly sensitive or “as suspicious as Lin Daiyu,” drawing a culturally relevant analogy. Non-critical fluctuations would also be reflected through real-time changes in their status description but without the red alert, ensuring a continuous stream of feedback. Crucially, the system was biased towards providing positive reinforcement for improvements, fostering an overall supportive and encouraging environment.

### 2.7 Participants

This study was initially launched in the autumn semester of 2022, emerging from an urgent need to dynamically monitor student mental health during a campus-wide lockdown due to the COVID-19 pandemic in Hohhot. The traditional, infrequent survey-based assessments were deemed insufficient for the volatile psychological environment. Consequently, this research began as an enhanced mental health management initiative for a specific cohort of students.

A total of 177 undergraduate students (98 female, 79 male; mean age = 20.53 years) from four classes in their second (sophomore) and third (junior) years of a clinical medicine program at a medical university participated in the study. The sampling method employed was **cluster sampling**, as the initial cohort consisted of all students residing in a single dormitory building under unified management during the lockdown. All students in this building were initially enrolled in the program. The final sample of 177 participants represents those students who remained and provided continuous, complete data across the entire one-and-a-half-year (three academic semesters) study period. Students who left the cohort prematurely (e.g., due to changes in lockdown status or academic leave) were excluded from the final analysis due to incomplete longitudinal data.

The sample size was determined by the size of the accessible student cluster in the unique real-world context, rather than by a priori power analysis, which is appropriate for the exploratory nature of this study. Following detailed introductions by their life guidance teachers (counselors), all participants provided voluntary informed consent electronically via the WeChat mini-program, fully understanding the research objectives, procedures, and confidentiality measures. The demographic characteristics of the participants are detailed in Table 2.

**Table 2:** Participant characteristics.

Class	Female	Male	Total	Average age
A	31	21	52	20.60
B	25	22	47	20.34
C	18	18	36	20.64
D	24	18	42	20.55
All classes	98	79	177	20.53

#### 2.7.1 Ethical statement

The study protocol was approved by the Ethics Committee of Inner Mongolia People’s Hospital as part of a funded research project (Approval No. 2022LL019, Date: March 10, 2022). The research was conducted in accordance with the Declaration of Helsinki, ensuring voluntary participation and data security.

### 2.8 Measurement process

The data collection for this longitudinal study officially commenced in September 2022 and concluded in March 2024, spanning three academic semesters. Prior to the official start, the WeChat mini-program had been fully developed and had undergone preliminary small-scale testing. The measurement process consisted of three primary components:

1. **Periodic psychometric screening:** At the beginning of each semester, participants were organized to complete a systematic self-report screening using the full battery of standardized scales (SAS, SDS, SCL-90, EPQ, 16PF, and MBTI). This provided periodic baseline data for each assessment wave.
2. **Continuous ecological data collection:** Throughout the one-and-a-half-year period, we implemented weekly data collection tasks via the mini-program. These tasks included “cognitive voting” and “behavioral check-ins,” designed to encourage students’ expression of their views and emotional attitudes related to daily life events and situations.
3. **Data collection summary:** Over the course of the study, a total of 87 distinct cognitive voting activities were conducted, achieving an average participation rate of 79%. Concurrently, 66 behavioral check-in tasks were assigned, with an average participation rate of 42%. The lower participation rate for behavioral check-ins is primarily attributed to their event-specific nature; some tasks targeted experiences (e.g., specific academic challenges or social events) that were only applicable to a subset of participants at any given time. Nevertheless, the majority of check-in tasks focused on common aspects of student life and learning, aiming to reflect general behavioral patterns.

This multi-modal, multi-wave data collection approach allowed for a comprehensive and dynamic monitoring of students’ mental health status, providing a rich dataset for subsequent analysis and intervention.

### 2.9 Statistical methods

All statistical analyses were conducted using Python (version 3.8.18) with the SciPy (version 1.10.1), Pandas (version 1.5.3), and Scikit-learn libraries. Data visualization was performed using Matplotlib (version 3.7.1) and Seaborn (version 0.12.2). The level of statistical significance for all tests was set at p<0.05. The analysis was designed to address two core research questions.

#### 2.9.1 Analysis of assessment accuracy

The first objective was to evaluate whether the dynamic assessment model could more accurately reflect students’ mental health status compared to traditional static scales, using professionally administered Hamilton Rating Scales (HAM-A and HAM-D) as the clinical gold standard. The analysis varied based on the nature of the outcome.

- **For categorical classification performance (anxiety/depression):** To evaluate the ability of SAS and SDS scores (both traditional and dynamic) to correctly classify students’ clinical anxiety and depression status, we constructed Receiver Operating Characteristic (ROC) curves and calculated the Area Under the Curve (AUC). We also computed standard performance metrics including accuracy, recall, and F1-score.
- **For continuous score prediction (general symptoms):** To assess the performance of SCL-90 scores in predicting continuous HAM-A and HAM-D scores, we used two primary metrics: the coefficient of determination (R-squared, $R^{2}$) to quantify the proportion of variance explained, and the Mean Squared Error (MSE) to measure prediction error.
- **For agreement analysis:** To assess the agreement between traditional and dynamically adjusted scores, we performed both Pearson (for linear relationships) and Spearman (for monotonic relationships) correlation analyses. Given that Shapiro- Wilk tests indicated that most of our data did not strictly adhere to a normal distribution (p<0.05), the non-parametric Spearman correlation provides a more robust measure of association.

#### 2.9.2 Analysis of intervention effects

The second objective was to explore the potential intervention effect of long-term engagement with the dynamic assessment system. This was analyzed through a quasi-experimental design.

- **Comparison group:** The primary intervention group consisted of the 177 students who actively participated in our dynamic assessment program. A comparison group was established, comprising 92 students from two other classes of the same grade and university who were not managed by our hospital. This group only completed the routine, semester-based self-report scales (SAS, SDS, and SCL-90) and did not participate in the dynamic assessment or interactive components of the mini-program.
- **Statistical tests:** We calculated the change score (ΔScore) for each participant in both groups between the first (October 2022) and final (March 2024) assessments.
  ○ **Independent-sample *t*-tests** were used to compare the mean ΔScore between the intervention and comparison groups to determine if our program led to significantly greater improvements.
  ○ **Multivariate analysis of variance (MANOVA)** was employed to simultaneously evaluate the overall impact of the intervention across multiple mental health dimensions (e.g., anxiety, depression, somatization).
  ○ We acknowledged that prerequisite assumptions for parametric tests, such as normality and homogeneity of variances (Levene’s test), were not always met. However, given the robustness of these tests with larger sample sizes, and supplemented by our non-parametric analyses, we proceeded while interpreting the results with appropriate caution.
- **Effect size analysis:** To evaluate the practical significance of any observed changes, we calculated Cohen’s *d* for all key comparisons, providing a standardized measure of the magnitude of the intervention effect.

## 3 Results

### 3.1 Comparison of SAS assessment results

#### 3.1.1 Descriptive and inferential statistics

**Figure 9:**
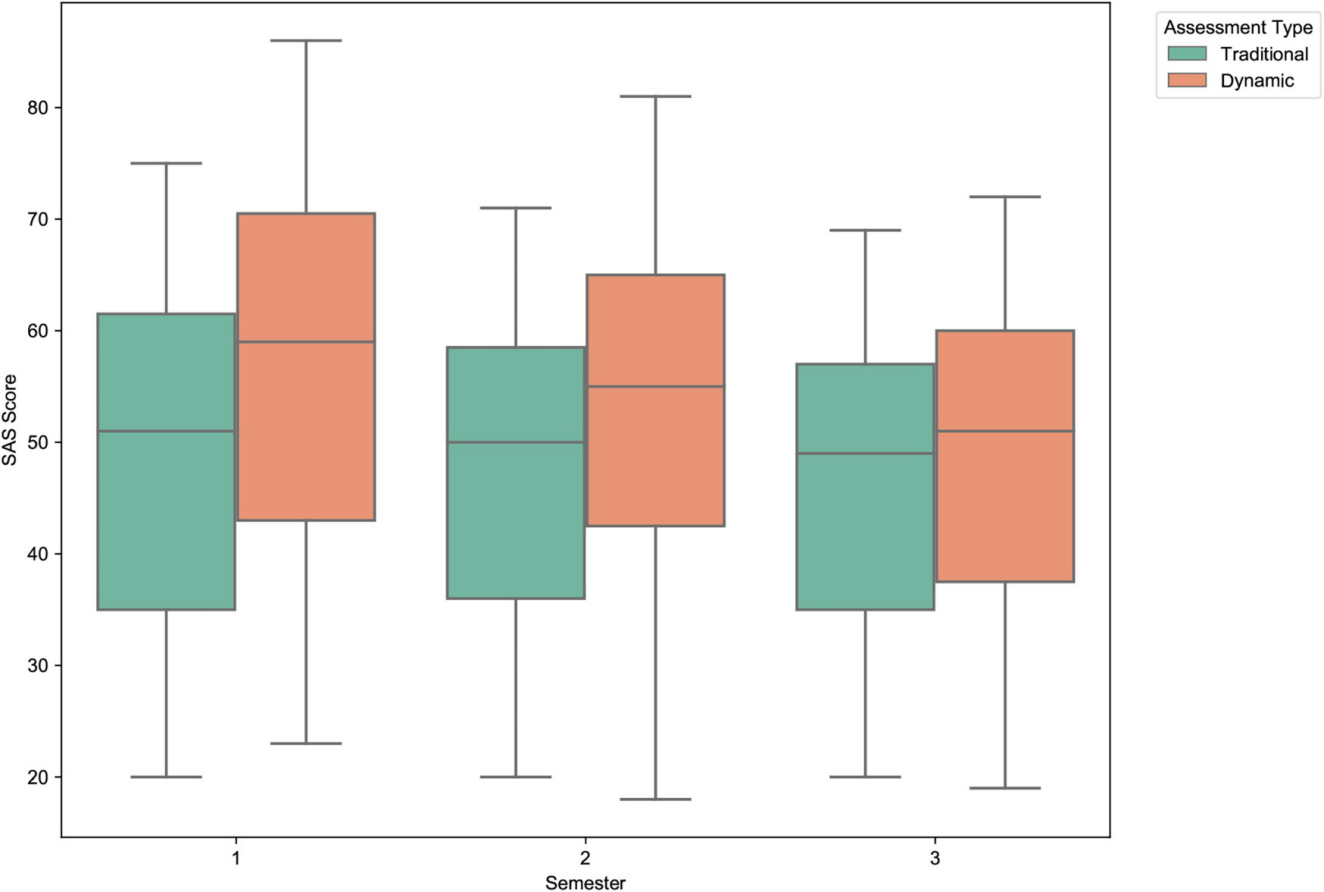
Distribution of traditional vs. dynamic SAS scores across semesters. The boxplots illustrate a consistent downward trend in anxiety scores for both assessment types. Notably, the dynamically adjusted scores show a more pronounced decrease over time and a reduction in score variance by the third semester, suggesting a positive longitudinal effect and greater score stabilization.

As illustrated in Figure 9, both traditional and dynamically corrected Self-Rating Anxiety Scale (SAS) scores exhibited a gradual downward trend across the three semesters of the study. The mean score for the traditional assessment decreased from 48.77 (Semester 1) to 46.56 (Semester 3). In contrast, the dynamic assessment showed a more substantial reduction, from an initial mean of 56.78 to 48.67 over the same period.

Inferential statistics, detailed in Table 3, confirm these observations. Paired-sample *t*-tests revealed that the decrease in scores between semesters was statistically significant for both methods (p<.001 for all comparisons). Furthermore, the differences between the traditional and dynamically corrected scores were statistically significant within each of the three semesters (p<.001 for all), indicating that our dynamic adjustment algorithm consistently and significantly altered the assessment results.

**Table 3:** Comparison of SAS scores across semesters with *t*-test results.

Assessment type	Mean SAS score by semester			Paired-sample *t*-test (sem1 vs. sem3)		
	Sem 1	Sem 2	Sem 3	Mean diff.	95% CI	*p*-value
SAS traditional	48.77	47.47	46.56	−2.21	[−2.67, −1.75]	<.001
SAS dynamic	56.78	52.85	48.67	−8.10	[−9.18, −7.02]	<.001
*t*-test: T vs. D	p<.001	p<.001	p<.001			

#### 3.1.2 Model evaluation metrics

To evaluate the predictive validity of the dynamic model against the clinical gold standard (HAM-A), we assessed its performance in both the first and third semesters. As detailed in the combined summary in Table 4, the dynamic correction model demonstrated consistently superior performance across both time points. In the first semester, the accuracy of the dynamic model was 94.86%, a significant improvement over the traditional model’s 77.14%, with a corresponding increase in recall from 75.00% to 96.43%. This high level of performance was sustained into the third semester (accuracy: 92.00% vs. 83.43%), suggesting that the dynamic adjustment offers greater consistency and reliability for long-term tracking.

**Table 4:** Prediction performance metrics of SAS scores in first and third semesters.

Metric	First semester		Third semester	
	SAS traditional	SAS dynamic	SAS traditional	SAS dynamic
Accuracy	77.14%	94.86%	83.43%	92.00%
Recall	75.00%	96.43%	76.70%	89.32%
F1 score	0.81	0.96	0.84	0.93

**Figure 10:**
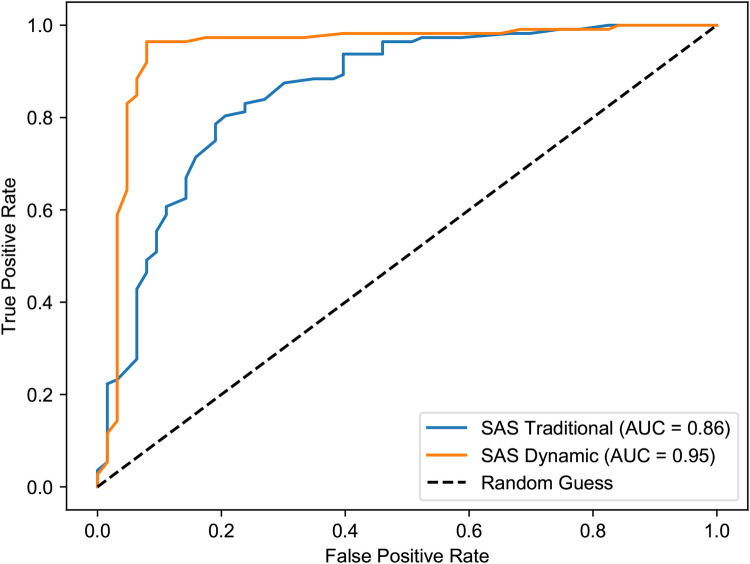
ROC curves for SAS vs. HAM-A anxiety in the first semester. The graph clearly shows the superior discriminative ability of the dynamic assessment model (AUC = 0.95) compared to the traditional model (AUC = 0.86) in identifying clinically significant anxiety.

**Figure 11:**
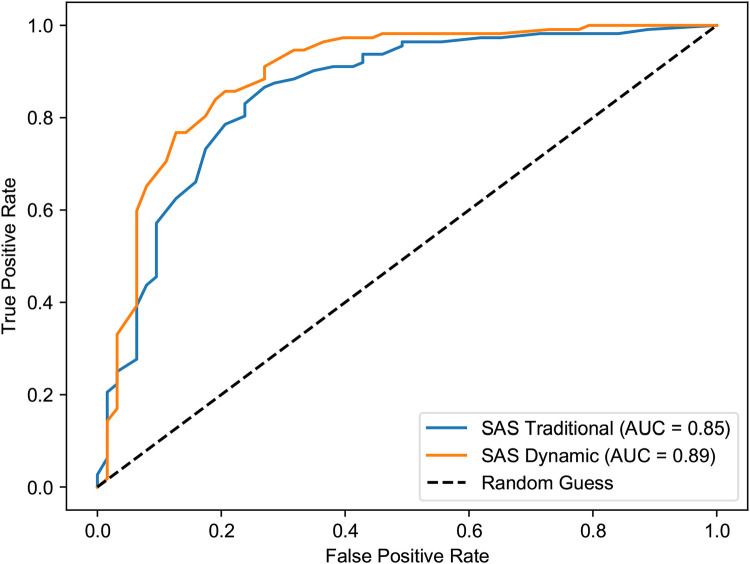
ROC curves for SAS vs. HAM-A anxiety in the third semester. The dynamic model maintains its predictive advantage (AUC = 0.89) over the traditional model (AUC = 0.85), indicating sustained performance in long-term tracking.

The superiority of the dynamic model is further evidenced by the Receiver Operating Characteristic (ROC) curve analysis (Figures 10, 11). In both the first and third semesters, the Area Under the Curve (AUC) for the dynamically corrected results was consistently higher (Semester 1: AUC = 0.95 vs. 0.86; Semester 3: AUC = 0.89 vs. 0.85), demonstrating a stronger and more accurate correlation with the clinician-rated HAM-A assessment results.

### 3.2 Comparison of SDS assessment results

#### 3.2.1 Descriptive and inferential statistics

**Figure 12:**
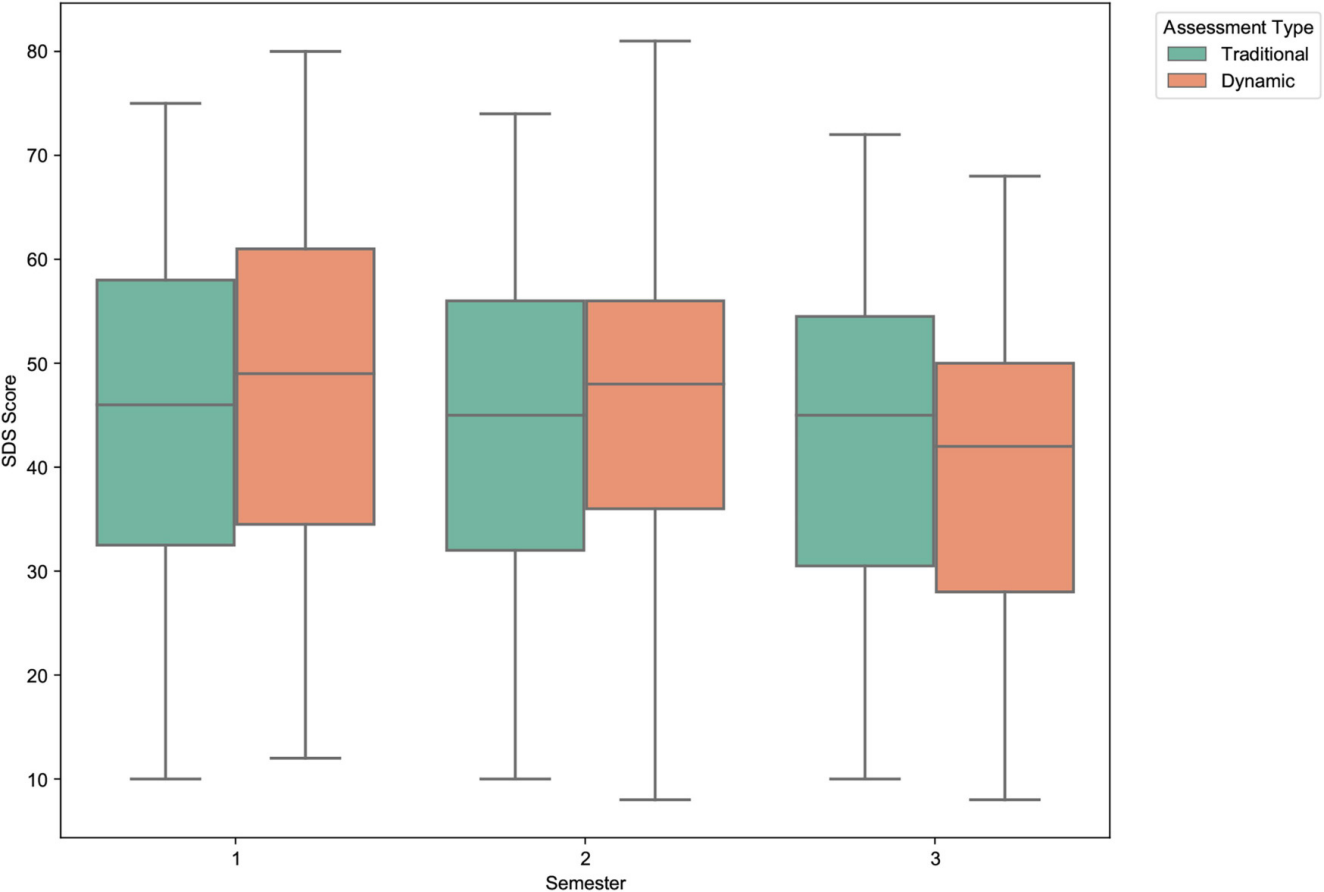
Distribution of traditional vs. dynamic SDS scores across semesters. The boxplots highlight a much steeper decline in depression scores for the dynamic assessment compared to the relatively stable traditional scores. This suggests the dynamic model is more sensitive to longitudinal changes in depressive symptoms.

Similar to the anxiety scores, the Self-Rating Depression Scale (SDS) scores also showed a general decline over the study period, with the dynamic model again demonstrating a more pronounced effect (Figure 12). The mean score for the traditional assessment remained relatively stable, decreasing slightly from 44.43 (Semester 1) to 42.91 (Semester 3). In stark contrast, the dynamically adjusted scores decreased markedly from a mean of 47.27 in the first semester to 39.78 in the third.

The statistical significance of these trends is confirmed in Table 5. While the longitudinal decrease in the traditional SDS scores was statistically significant (*p* = .019), the magnitude of change in the dynamic scores was far greater and more significant (p<.001). Paired-sample *t*-tests also showed significant differences between the traditional and dynamic scores in the first and third semesters, underscoring the substantial impact of our real-time data integration.

**Table 5:** Comparison of SDS scores across semesters with *t*-test results.

Assessment type	Mean SDS score by semester			Paired-sample *t*-test (Sem1 vs. Sem3)		
	Sem 1	Sem 2	Sem 3	Mean diff.	95% CI	*p*-value
SDS traditional	44.43	44.11	42.91	−1.52	[−2.82, −0.22]	.019
SDS dynamic	47.27	45.81	39.78	−7.49	[−8.87, −6.11]	< .001
*t*-test: T vs. D	p=.002	p=.054	p<.001			

#### 3.2.2 Model evaluation metrics

The dynamic model’s superior ability to predict clinically-rated depression (HAM-D) was evident across the study. As summarized in Table 6, the dynamic model consistently outperformed the traditional scale. In the first semester, the dynamic model achieved an accuracy of 92.57% and a recall of 85.71%, substantially higher than the traditional model’s 73.71% and 60.71%, respectively. This enhancement is critical, as it indicates a marked reduction in false negatives (missed cases). This superior performance was maintained in the third semester, with the dynamic model showing higher accuracy (94.29% vs. 81.14%) and recall (78.26% vs. 67.39%).

**Table 6:** Prediction performance metrics of SDS scores in first and third semesters.

Metric	First semester		Third semester	
	SDS traditional	SDS dynamic	SDS traditional	SDS dynamic
Accuracy	73.71%	92.57%	81.14%	94.29%
Recall	60.71%	85.71%	67.39%	78.26%
F1 score	0.69	0.92	0.65	0.88

**Figure 13:**
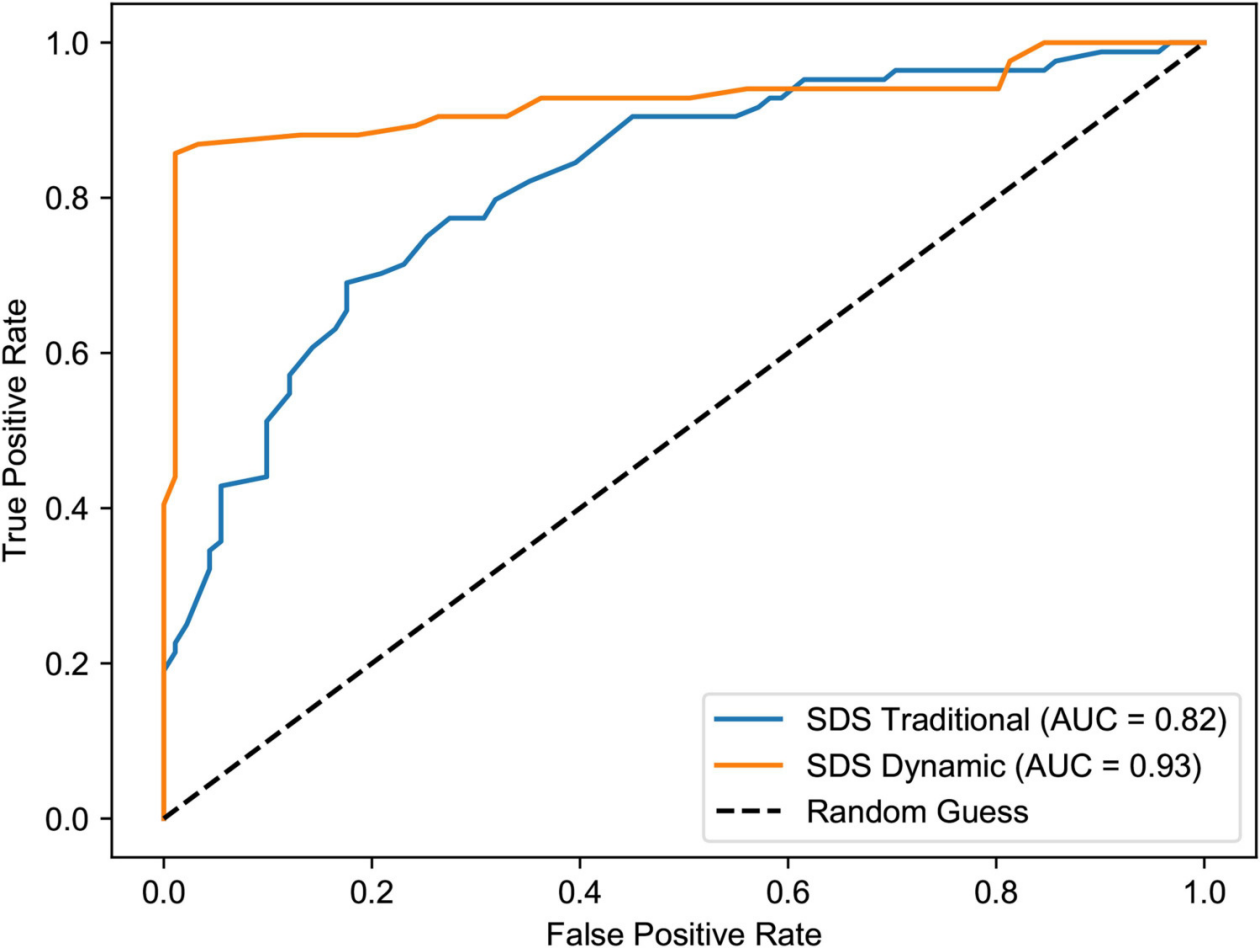
ROC curves for SDS vs. HAM-D depression in the first semester. The dynamic model (AUC = 0.93) demonstrates a substantially improved ability to discriminate between clinical and non-clinical depression compared to the traditional model (AUC = 0.82).

**Figure 14:**
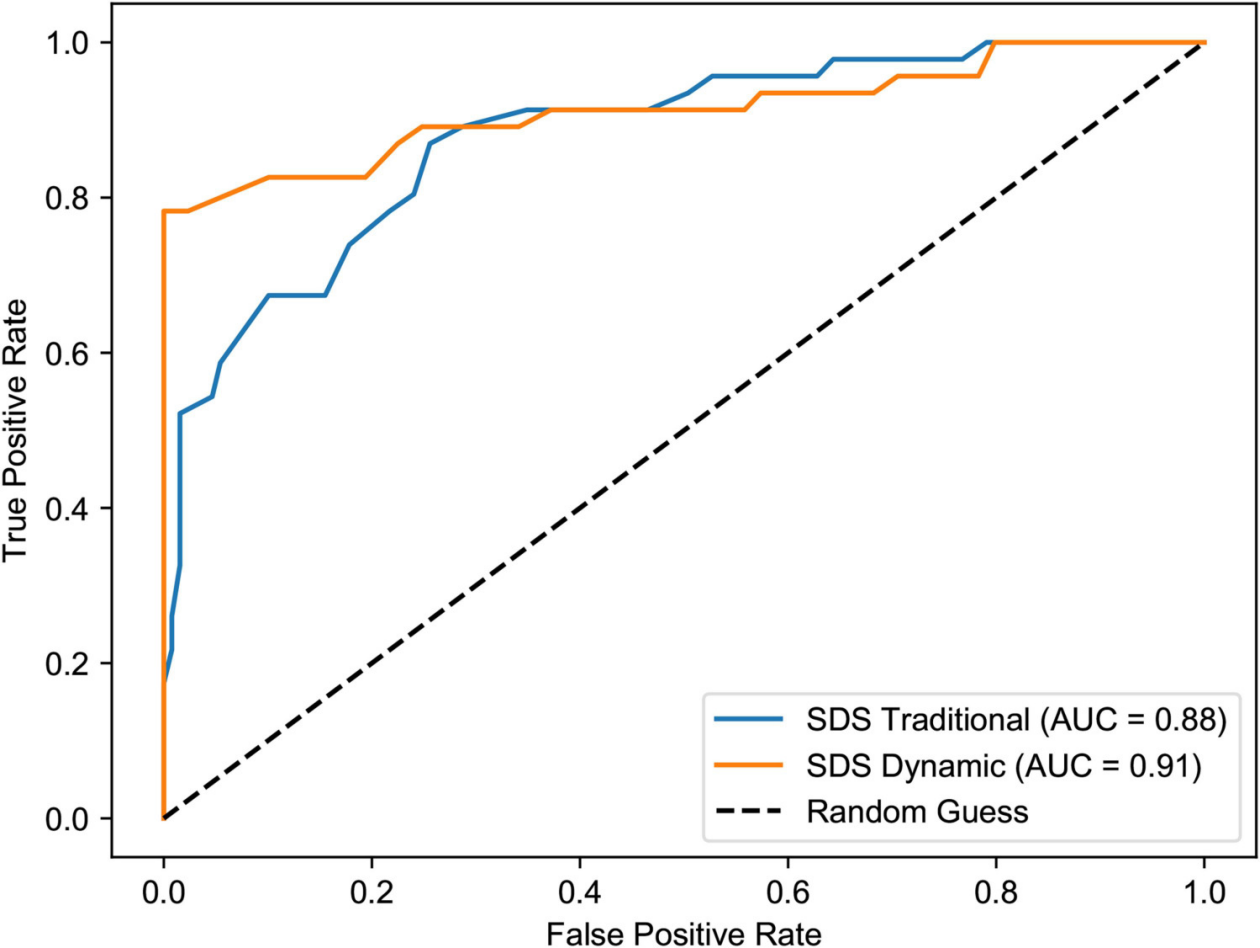
ROC curves for SDS vs. HAM-D depression in the third semester. The dynamic model continues to show superior performance (AUC = 0.91) over the traditional model (AUC = 0.88), reinforcing its value in long-term assessment.

This enhanced predictive power is also clearly visualized in the ROC curve analysis (Figures 13, 14). The dynamic model consistently yielded a higher AUC in both the first semester (0.93 vs. 0.82) and the third semester (0.91 vs. 0.88), confirming its stronger correlation with and predictive accuracy for the HAM-D assessment results.

### 3.3 Comparison of SCL-90 assessment results

#### 3.3.1 Descriptive and inferential statistics

**Figure 15:**
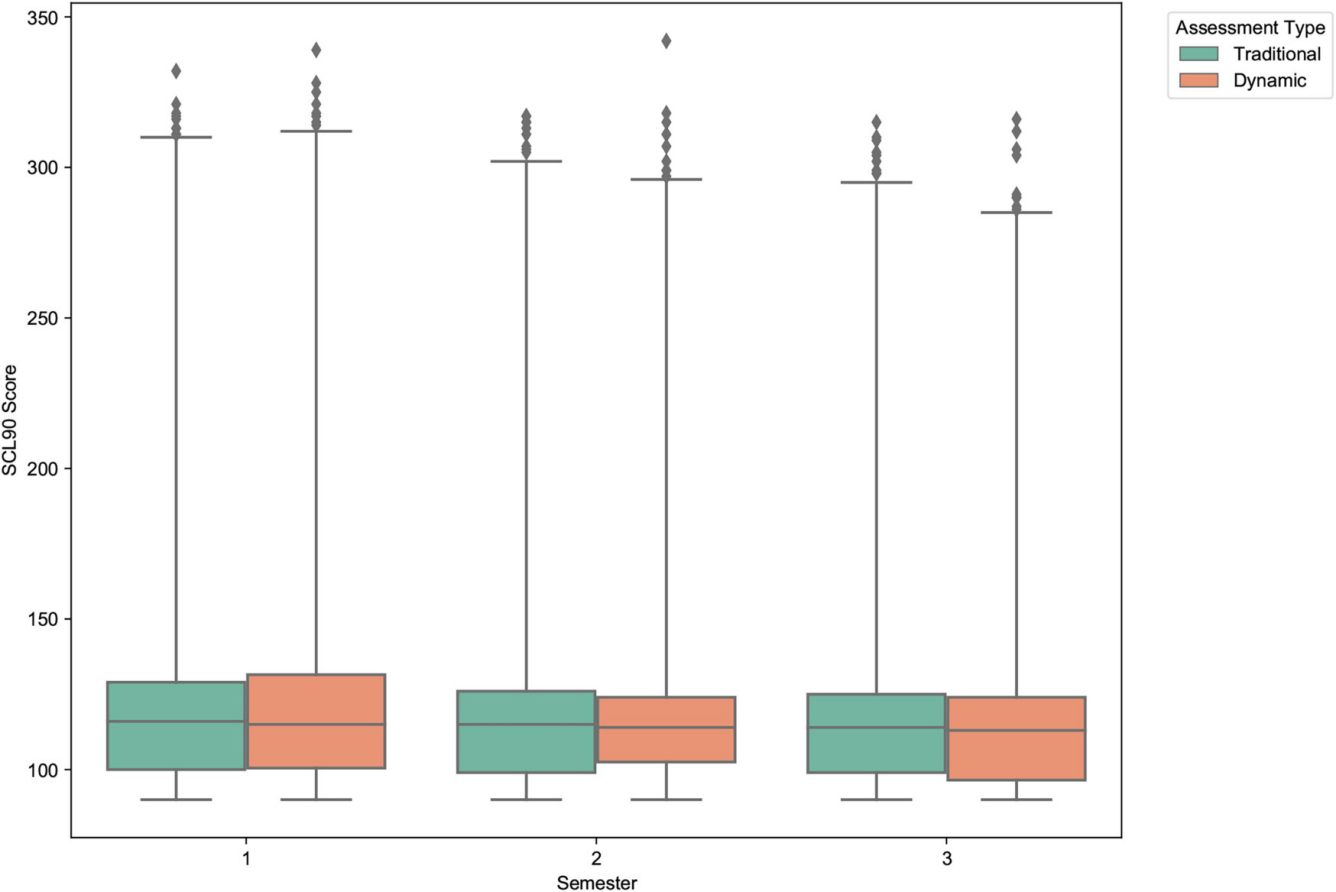
Distribution of traditional vs. dynamic SCL-90 scores across semesters. The boxplots show a general decline in overall psychological symptoms. The decrease is more statistically significant and substantial for the dynamically adjusted scores, indicating greater sensitivity to longitudinal changes.

The analysis of the Symptom Checklist-90 (SCL-90) total scores reveals a similar longitudinal trend, as shown in Figure 15. Both assessment methods registered a decrease in overall psychological symptomatology over the three semesters. The traditional SCL-90 total score showed a modest, non-significant decrease from a mean of 146.80 to 132.31. In contrast, the decrease in the dynamically adjusted scores was more pronounced and statistically significant, dropping from 150.14 in Semester 1 to 123.31 in Semester 3 (p = .019), as detailed in Table 7. This suggests that the dynamic model was more sensitive to overall improvements in students’ self-reported psychological well-being over time.

**Table 7:** Comparison of SCL-90 scores across semesters with *t*-test results.

Assessment type	Mean SCL-90 score by semester			Paired-sample *t*-test (Sem1 vs. Sem3)		
	Sem 1	Sem 2	Sem 3	Mean diff.	95% CI	*p*-value
SCL-90 traditional	146.80	139.13	132.31	−14.49	[−30.34, 1.36]	.073
SCL-90 dynamic	150.14	133.43	123.31	−26.83	[−48.11, −5.55]	.019
*t*-test: T vs. D	p=.574	p=.278	p=.031			

#### 3.3.2 Model evaluation metrics

The evaluation of the SCL-90 model’s performance in predicting continuous HAM-A and HAM-D scores revealed a more complex pattern, as detailed in Tables 8, 9.

**Table 8:** Top 3 SCL-90 predictors for HAM-A and HAM-D in the first semester.

Predictors rank	Traditional SCL-90			Dynamic SCL-90		
Predicting HAM-A	$R^{2}$	MSE	Predicting HAM-A	$R^{2}$	MSE
1	Other	0.21	139.15	Total score	0.34	116.99
2	Total score	0.20	141.34	Obsessive	0.33	118.06
3	Depression	0.20	142.38	Phobia	0.33	119.32
Predictors rank	Predicting HAM-D	$R^{2}$	MSE	Predicting HAM-D	$R^{2}$	MSE
1	Obsessive	0.07	200.63	Anxiety	0.28	154.93
2	Depression	0.07	200.90	Somatization	0.28	155.74
3	Phobia	0.07	201.14	Phobia	0.28	156.82

**Table 9:** Top 3 SCL-90 predictors for HAM-A and HAM-D in the third semester.

Rank	Traditional SCL-90			Dynamic SCL-90		
Predicting HAM-A	$R^{2}$	MSE	Predicting HAM-A	$R^{2}$	MSE
1	Depression	0.17	110.79	Psychoticism	0.17	111.91
2	Total score	0.17	110.88	Anxiety	0.16	112.06
3	Anxiety	0.17	111.24	Total score	0.16	113.27
Rank	Predicting HAM-D	$R^{2}$	MSE	Predicting HAM-D	$R^{2}$	MSE
1	Obsessive	0.25	84.17	Psychoticism	0.08	102.74
2	Total score	0.21	87.88	Somatization	0.08	103.22
3	Interpersonal	0.21	88.01	Obsessive	0.07	103.59

In the first semester, the dynamic model demonstrated a stronger correlation with the clinical ratings. For instance, when predicting HAM-A scores, the dynamic model’s total score achieved an R-squared of 0.34, substantially higher than the traditional model’s R-squared of 0.20. This indicates that the dynamic scores initially explained a larger proportion of the variance in clinically-rated anxiety. The dynamic model also showed a lower Mean Squared Error (MSE) for predicting HAM-A (116.99 vs. 141.34), suggesting a higher initial prediction accuracy.

However, this advantage diminished by the third semester. While the traditional model’s predictive power for HAM-D (as measured by the obsessive-compulsive subscale) showed a relatively strong correlation ($R^{2}$ = 0.25), the dynamic model’s correlations weakened across most dimensions (e.g., psychoticism predicting HAM-D, $R^{2}$ = 0.08). Furthermore, the prediction error for the dynamic model increased for HAM-D (MSE = 102.74) compared to the traditional model (MSE = 84.17). This suggests that while the dynamic adjustments are effective for simpler constructs like anxiety and depression, their specificity may decrease over time for the multi-faceted SCL-90, potentially due to interference from complex life events not captured by our model.

### 3.4 Comparison of HAM-A and HAM-D pre- and post-assessments

To assess the potential intervention effect of long-term participation in the dynamic assessment system, we analyzed the changes in clinician-rated Hamilton Anxiety (HAM-A) and Hamilton Depression (HAM-D) scores from the first semester (November 2022) to the third semester (March 2024).

**Figure 16:**
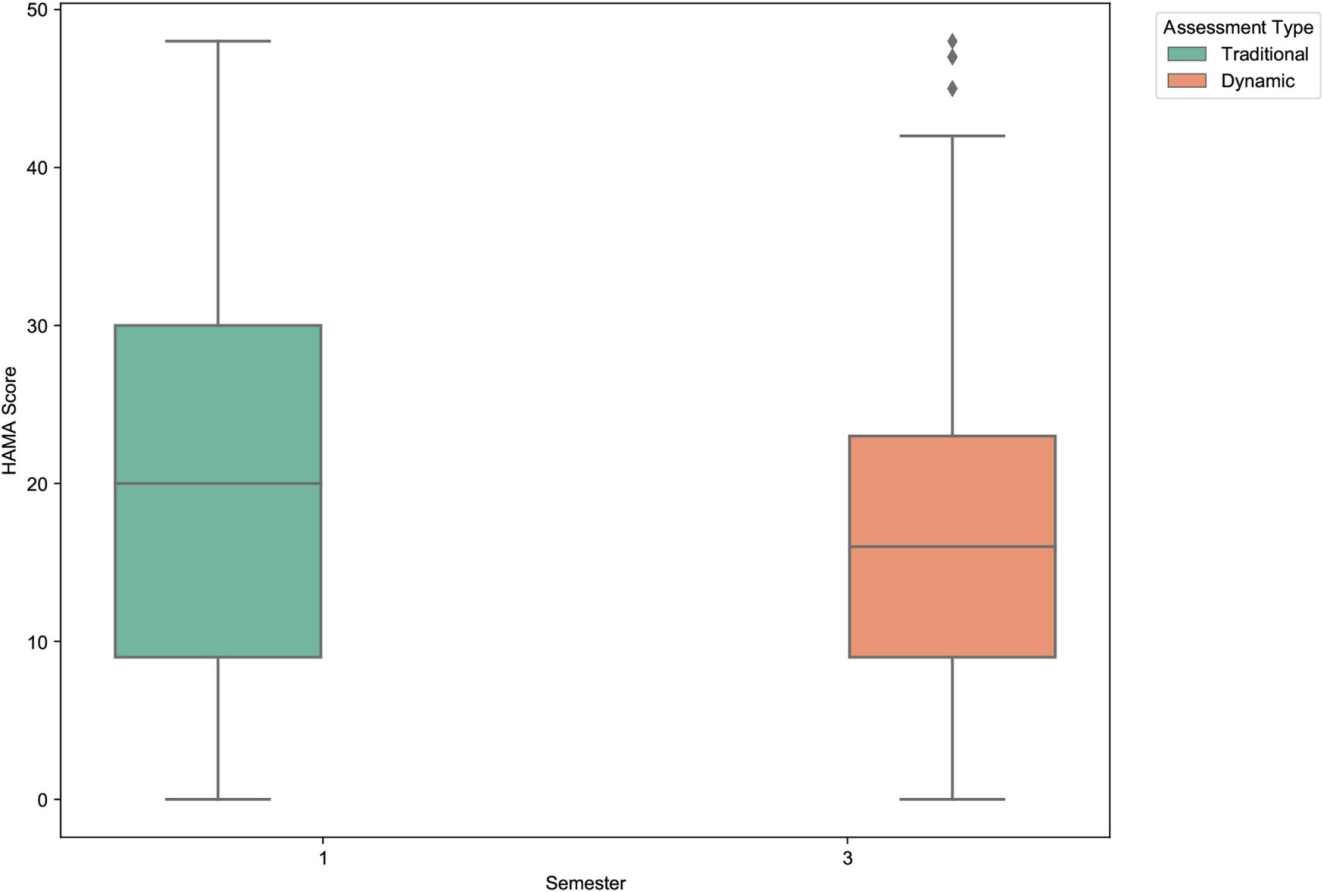
Clinician-rated anxiety (HAM-A) score comparison between first and third semesters. The boxplot shows a statistically significant decrease in the mean anxiety scores among students who participated in the dynamic assessment system over one and a half years.

**Figure 17:**
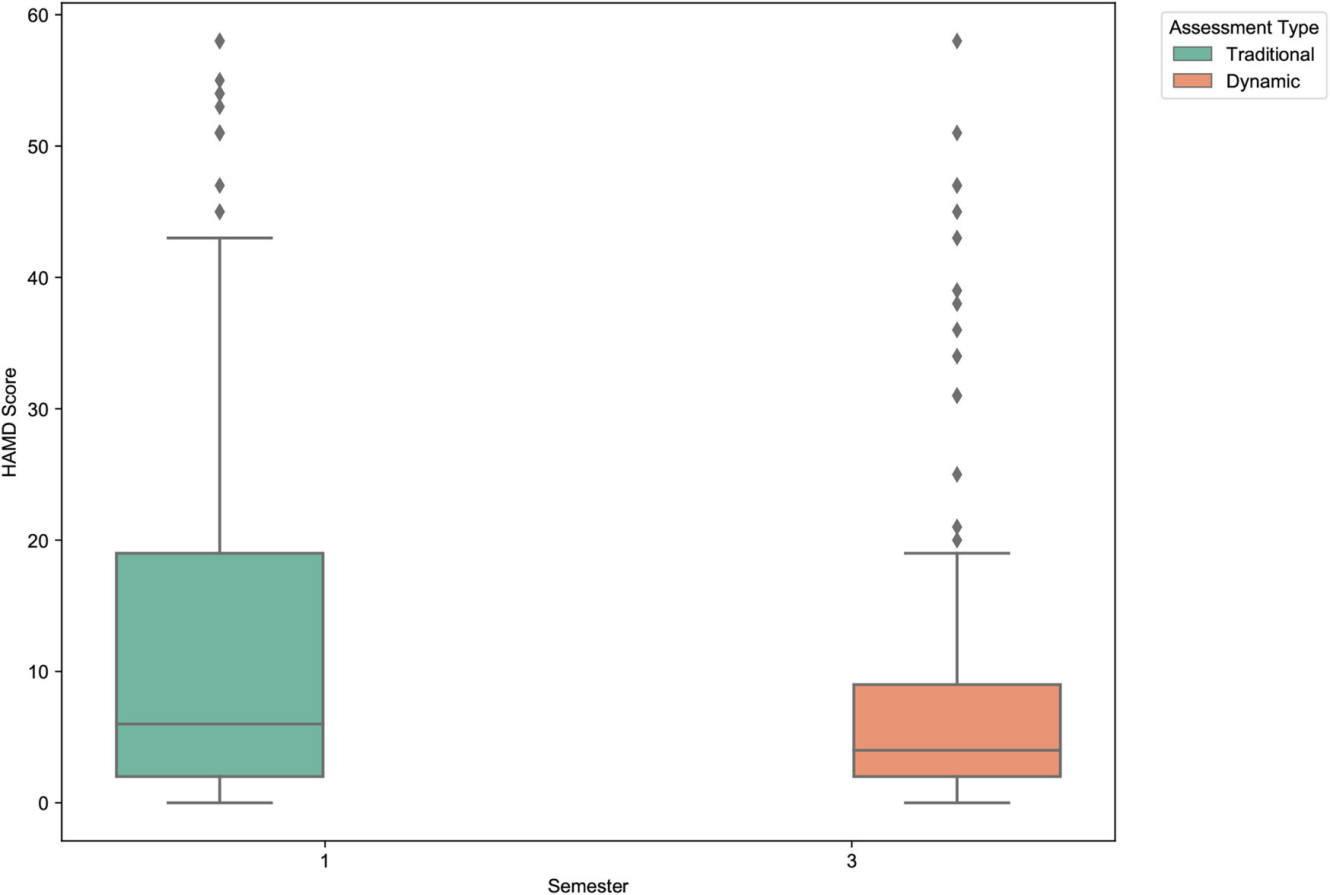
Clinician-rated depression (HAM-D) score comparison between first and third semesters. A pronounced and statistically significant reduction in mean depression scores is observed, highlighting the potential therapeutic benefit of the dynamic assessment and feedback system.

As shown in Table 10 and visualized in Figures 16, 17, participating students exhibited statistically significant reductions in both anxiety and depression levels over the study period. The mean HAM-A score decreased by 15.2%, from 20.61 to 17.49. The mean HAM-D score showed an even more pronounced reduction of 40.0%, decreasing from 13.02 to 7.81. Paired-sample *t*-tests confirmed that both of these changes were statistically significant (HAM-A: *p* = .004; HAM-D: p<.001).

**Table 10:** Comparison of HAM-A and HAM-D scores between first and third semesters.

Scale	Mean score		Paired-sample *t*-test (Sem1 vs. Sem3)		
	Sem 1	Sem 3	Mean diff.	95% CI	*p*-value
HAM-A	20.61	17.49	−3.12	[−5.25, −1.00]	.004
HAM-D	13.02	7.81	−5.21	[−7.71, −2.71]	<.001

These findings are particularly noteworthy as they are based on independent clinical assessments rather than self-report measures. The results suggest that continuous engagement with the dynamic assessment system—which provides regular feedback and promotes self-monitoring—may have a tangible, positive therapeutic effect. This demonstrates the system’s potential not only as a more accurate measurement tool but also as a practical, scalable intervention for improving student mental health.

## 4 Discussion

This study introduced and evaluated an AI-driven dynamic psychological assessment system designed to correct and enhance traditional mental health scales using daily behavioral and cognitive data from university students. Our primary findings robustly support the feasibility and superiority of this novel approach. The results demonstrate that the dynamic model significantly improved the accuracy of anxiety (SAS) and depression (SDS) assessments compared to static, single-time-point measurements, showing a stronger correlation with clinician-rated gold standards. Furthermore, long-term engagement with the system was associated with significant reductions in clinically-rated anxiety (HAM-A) and depression (HAM-D) scores, suggesting the system’s potential not only as a measurement tool but also as an integrated assessment-intervention loop. However, the study also revealed challenges, particularly in the declining predictive power of the model for the more complex, multi-faceted SCL-90 scale over time. In the following sections, we will interpret these principal findings, discuss their clinical and theoretical implications, analyze the model’s performance limitations, and outline the study’s limitations and future research directions.

### 4.1 Principal findings and clinical implications

The central finding of this study is that integrating high-frequency, ecologically-valid data from students’ daily lives significantly enhances the accuracy of traditional psychometric scales for anxiety and depression. The superior performance of our dynamic model, evidenced by higher AUCs and a stronger correlation with clinician ratings, underscores a fundamental limitation of static assessments: their inability to capture the fluid, context-dependent nature of mental states. A single questionnaire provides a cross-sectional “snapshot,” which can be easily biased by a student’s mood on a particular day. In contrast, our dynamic system functions like a longitudinal “film,” continuously updating the assessment based on a stream of cognitive and behavioral data points, thereby creating a more robust, nuanced, and authentic psychological profile.

The clinical implications of this finding are profound, signaling a potential paradigm shift in mental health assessment. In an era where daily life is increasingly mediated by digital technology, platforms like WeChat or wearable devices are becoming vast, untapped repositories of behavioral and cognitive data. Our research provides a proof-of-concept that these digital footprints can be ethically harnessed to move beyond reactive, clinic-based assessments towards a model of proactive, continuous, and personalized mental health monitoring. While the tools developed in this study are still nascent, they point towards a future where “digital phenotyping” could become a cornerstone of clinical practice. Such systems could enable clinicians to detect subtle negative changes in a student’s state long before they escalate into a crisis, facilitating early and targeted interventions.

Furthermore, the significant reduction in clinically-rated HAM-A and HAM-D scores suggests that the assessment process itself can be therapeutic. By engaging students in daily self-monitoring and providing immediate, narrative-based feedback, our system creates an active assessment-intervention loop. This process aligns with the principles of measurement-based care, where continuous data is used to inform and guide treatment. The gamified and humanized feedback encourages self-reflection and may empower students to make small but meaningful adjustments to their daily routines and thought patterns, fostering a sense of agency over their own mental well-being. This suggests that the future of digital mental health tools lies not just in their predictive power, but in their ability to function as interactive companions that promote psychological resilience.

### 4.2 Interpretation in light of theoretical frameworks

While this study was initially driven by practical needs rather than a single theoretical doctrine, its design and findings resonate strongly with several contemporary psychological frameworks. Our approach can be understood as an applied synthesis of principles rooted in the state-trait theory of personality, Ecological Momentary Assessment (EMA), and the emerging field of digital phenotyping.

At its core, our research directly addresses the classic **state-trait distinction** (1). Our fundamental premise is that traditional scales, while effective at capturing stable “traits,” are insufficient for tracking the moment-to-moment “states” that constitute daily emotional life. By continuously integrating daily cognitive and behavioral data, our dynamic model is a deliberate attempt to create a “state-sensitive” measurement tool. The finding that our dynamic scores are more predictive of clinical ratings supports the idea that an accumulation of state-level data provides a more valid picture of an individual’s current mental health status than a single trait-level snapshot.

The methodology itself is a direct application of **Ecological Momentary Assessment (EMA)**. By collecting high-frequency data in the students’ natural environment, we mitigate the recall bias inherent in retrospective questionnaires. This ecological validity is crucial for understanding the real-world triggers and fluctuations of anxiety and depression. Furthermore, our work extends EMA by not just collecting data, but by using it to actively correct and inform psychometric scores in near real-time.

Finally, our study contributes to the burgeoning field of **digital phenotyping**. The ultimate vision, as articulated in literature on the future of psychometrics, is the use of multimodal data from daily life—captured via smartphones and wearable devices—to construct a comprehensive picture of mental well-being (11). Our WeChat mini-program serves as a direct, albeit early, implementation of this vision. By translating digital interactions (cognitive votes and behavioral check-ins) into psychometrically meaningful adjustments, we are building a data-driven “digital phenotype” of student mental health. The finding that this process may also be therapeutic aligns with theories of **Self-Regulation**, where the feedback from one’s own data is a critical mechanism for behavioral change and goal attainment.

### 4.3 Understanding the model’s performance over time

A particularly noteworthy and complex finding of this study was the observed decline in the predictive power of our dynamic model for the SCL-90 over time. While the model initially showed a clear advantage over the traditional scale in predicting clinical ratings, this superiority diminished by the third semester. We propose that this phenomenon is not attributable to a single cause, but rather to a multifactorial interplay of methodological, psychological, and contextual factors.

First, the possibility of a **reactivity effect** cannot be discounted. Over the course of one and a half years, it is plausible that students became “assessment-savvy.” They may have discerned the underlying patterns of the system, consciously or unconsciously adjusting their responses in cognitive voting and behavioral check-ins to present themselves in a more favorable light or to “manage” their profiles. This form of learned behavior would introduce noise into the ecological data, decoupling it from their authentic psychological state and thereby weakening the model’s predictive accuracy.

Second, the decline may reflect the inherent **limitations of our model in capturing the complexity of the SCL-90 construct and real-world life events**. The SCL-90 measures a broad spectrum of psychopathological symptoms, which are often influenced by significant, discrete life events (e.g., academic failures, relationship breakdowns, the end of the COVID-19 lockdown). Our model, while effective at tracking the general ebb and flow of anxiety and depression, may lack the specificity to account for the impact of such major external shocks on more complex symptom dimensions like psychoticism or paranoia. The initial success of the model might have occurred during a period of relative environmental stability (i.e., the lockdown), while its later decline could coincide with a return to a more chaotic and unpredictable post-pandemic campus life, where a wider range of unmeasured confounding variables came into play.

Third, and perhaps counterintuitively, the model’s declining predictive power might be a paradoxical signal of its **success as an intervention**. The continuous feedback and self-monitoring process may have genuinely enhanced students’ self-regulation and coping skills. As students became more psychologically resilient, their mental states may have stabilized and exhibited less variance. Consequently, their daily cognitive and behavioral patterns would become less predictive of pathology simply because there was less pathology to predict. In this view, the “signal” (i.e., symptom fluctuation) weakened, making it harder for the model to make accurate predictions against a baseline of improved mental health. Disentangling these three potential explanations presents a significant challenge and underscores a crucial direction for future research.

### 4.4 Limitations and future directions

Despite the promising findings, this study has several limitations that must be acknowledged. First, the research was conducted in the context of a unique historical event—the COVID-19 pandemic and subsequent campus lockdown. This “natural experiment” setting, while providing a compelling rationale for the study, introduces significant **confounding variables**. The observed improvements in student mental health could be partially attributed to the cessation of the lockdown and a return to normal campus life (*history effect*), or to the natural process of student maturation over the one-and-a-half-year period (*maturation effect*).

Second, our study design lacks a **randomized controlled trial (RCT)** framework. The comparison group, while similar in academic background, was not randomly assigned, which introduces potential selection bias. Furthermore, the act of being continuously monitored and engaged might have induced a **Hawthorne effect**, where participants’ behavior changed simply because they were aware of being studied. While our results provide strong preliminary evidence, a future RCT with randomized allocation to either the dynamic assessment group or a control group (receiving only traditional assessments) would be necessary to definitively establish the causal effect of our intervention.

Third, our data collection, while ecologically valid, was limited to self-report (cognitive votes) and semi-objective data (counselor-logged behavioral check-ins). Future research should aim to integrate more **objective, passive data streams**, such as smartphone sensor data (e.g., screen time, mobility patterns) or wearable device metrics (e.g., sleep patterns, heart rate variability). This would create a more comprehensive digital phenotype, reduce the potential for reactivity effects, and further enhance the model’s predictive power. Finally, the AI model itself, while effective, requires further refinement in its ability to interpret and adapt to complex, long-term psychological changes.

## 5 Conclusion

In conclusion, this study provides compelling evidence for the value of AI-driven dynamic psychological assessment as a means to correct and enhance traditional mental health scales. By integrating real-time behavioral and cognitive data, we developed a system that not only offers a more accurate and nuanced picture of university students’ mental health but also functions as a potential therapeutic tool through continuous, gamified feedback. Despite the inherent limitations of its quasi-experimental design, our research demonstrates a viable pathway towards a new paradigm of proactive, personalized, and continuous mental health care. This work underscores the immense potential of ethically harnessing digital technologies to move beyond static snapshots and create a more dynamic, responsive, and ultimately more effective system for supporting student well-being.

## Data Availability

The raw data supporting the conclusions of this article will be made available by the authors, without undue reservation.
